# DNA repair deficiency and senescence in concussed professional athletes involved in contact sports

**DOI:** 10.1186/s40478-019-0822-3

**Published:** 2019-11-14

**Authors:** Nicole Schwab, Karl Grenier, Lili-Naz Hazrati

**Affiliations:** 1 Department of Laboratory Medicine and Pathobiology, University of Toronto, 1 King’s College Cir, Toronto, ON M5S 1A8 Canada; 2 The Hospital for Sick Children, 555 University Ave, Toronto, ON M5G 1X8 Canada

**Keywords:** Traumatic brain injury, Senescence, Neurodegeneration, Ageing, Concussion

## Abstract

Mild traumatic brain injury (mTBI) leads to diverse symptoms including mood disorders, cognitive decline, and behavioral changes. In some individuals, these symptoms become chronic and persist in the long-term and can confer an increased risk of neurodegenerative disease and dementia diagnosis later in life. Despite the severity of its consequences, the pathophysiological mechanism of mTBI remains unknown. In this post-mortem case series, we assessed DNA damage-induced cellular senescence pathways in 38 professional athletes with a history of repeated mTBI and ten controls with no mTBI history. We assessed clinical presentation, neuropathological changes, load of DNA damage, morphological markers of cellular senescence, and expression of genes involved in DNA damage signaling, DNA repair, and cellular senescence including the senescence-associated secretory phenotype (SASP). Twenty-eight brains with past history of repeated mTBI history had DNA damage within ependymal cells, astrocytes, and oligodendrocytes. DNA damage burden was increased in brains with proteinopathy compared to those without. Cases also showed hallmark features of cellular senescence in glial cells including astrocytic swelling, beading of glial cell processes, loss of H3K27Me3 (trimethylation at lysine 27 of histone H3) and lamin B1 expression, and increased expression of cellular senescence and SASP pathways. Neurons showed a spectrum of changes including loss of emerin nuclear membrane expression, loss of Brahma-related gene-1 (BRG1 or SMARCA4) expression, loss of myelin basic protein (MBP) axonal expression, and translocation of intranuclear tau to the cytoplasm. Expression of DNA repair proteins was decreased in mTBI brains. mTBI brains showed substantial evidence of DNA damage and cellular senescence. Decreased expression of DNA repair genes suggests inefficient DNA repair pathways in this cohort, conferring susceptibly to cellular senescence and subsequent brain dysfunction after mTBI. We therefore suggest that brains of contact-sports athletes are characterized by deficient DNA repair and DNA damage-induced cellular senescence and propose that this may affect neurons and be the driver of brain dysfunction in mTBI, predisposing the progression to neurodegenerative diseases. This study provides novel targets for diagnostic and prognostic biomarkers, and represents viable targets for future treatments.

## Introduction

Traumatic brain injury (TBI) is a leading cause of death and disability worldwide, affecting an estimated 10 million individuals each year [62]. In particular mild TBI (mTBI), which includes concussions and sub-concussive blows to the head [16], affects the largest proportion of these individuals [10, 131]. mTBI, especially when experienced repetitively [52], is linked to several symptoms which are broad in nature, involving mood, behavior, and cognitive changes [126, 140]. Indeed, both acute and chronic symptoms of mTBI include headache, nausea, fatigue, confusion, irritability, and short-term memory loss [30, 104, 128, 160]. A majority of individuals only experience these symptoms acutely, recovering within weeks [98]. However, approximately 20% go on to experience these symptoms for longer than 3 months, at which point they are diagnosed with post-concussive syndrome (PCS) [59]. In addition to these symptoms, a history of mTBI has been associated with an increased risk of being diagnosed with dementia and/or a neurodegenerative disease [36, 44, 90], including Alzheimer’s disease (AD) [112], amyotrophic lateral sclerosis (ALS) [17], Parkinson’s disease (PD) [71], frontotemporal dementia (FTD) [129], and, more recently, chronic traumatic encephalopathy (CTE) [99, 101]. Currently, the pathophysiological mechanism driving brain dysfunction after mTBI, including lingering long-term symptoms and the propensity towards neurodegenerative disease, remains unknown.

Several of the clinical symptoms and pathological changes in mTBI are similar to those seen with cellular senescence. Senescence is defined as a state of permanent cell-cycle arrest and characterized by chronic inflammation through the secretion of pro-inflammatory chemokines, interleukins, and cytokines known collectively as senescence-associated secretory phenotype (SASP) factors [24, 37]. The accumulation of senescent cells in the brain is thought to drive ageing and age-related diseases [155], cognitive decline [6], and neurodegenerative pathology [81]. More recently, markers of senescence were shown to be elevated in a mouse model of mTBI [153] and, in our previous work, we have shown evidence of DNA damage in human cases with a history of acute and chronic mTBI [135].

Senescent cells normally accumulate with age [18], however senescence can also be induced prematurely in the context of chronic cellular stress [29]. Most notably, the accumulation of DNA damage in the form of double-stranded breaks is a potent inducer of cellular senescence [26]. The various forms of DNA damage accumulate with the natural ageing process, in part due to endogenous sources, such as metabolic reactive oxygen species (ROS), and partly due to exogenous agents such as radiation, alcohol and drug abuse, and UV light exposure [105]. Because cells are normally faced with these insults, they are equipped with an evolutionarily conserved endogenous repair pathway called the DNA damage response (DDR) [86]. The DDR is a large-scale, complex, and dynamic pathway which functions to restore integrity of DNA following a lesion [70]. Failure of the DDR to properly repair DNA results in the accumulation of DNA damage and subsequently cellular senescence, and as such it plays a crucial role in maintaining the genomic integrity and cellular function [92, 121, 168].

Deficiencies in DNA repair are known to underlie several neurological conditions in both humans and animal models [103, 147]. In fact, inefficient DNA repair has been proposed as an important factor in premature aging and the development of neurodegenerative diseases [93]. In AD, a two-fold increase in DNA damage has been found in the cortex compared to healthy controls [113]. Furthermore, AD brains have been reported as having decreased base-excision repair (BER) pathway activity [87]. Defects in DNA repair machinery have been linked to alpha-synuclein pathology and reduced dopaminergic innervation consistent with PD [136] and have been characterized in several neurodegenerative disorders including ALS and FTD [161]. DNA damage has therefore been suggested to play a role in age-related cognitive decline and pathology [11]. In fact, accumulation of DNA damage has been shown to predict progression from mild cognitive impairment (MCI) to AD prior to the emergence of any neuropathology [91].

Similarly, accumulation of senescent glial cells drives tau pathology and cognitive decline in mice [14]. Indeed, a mouse model of tau-dependent neurodegeneration accumulates p16 in astrocytes and microglia, and both pharmacogenetic and pharmacological clearance of these senescent cells alleviates tau hyperphosphorylation, gliosis, cortical and hippocampal degeneration, and improves cognitive function [14]. Consistent with this study, it was recently shown that human post-mortem brains with tau pathology presented with a senescence-associated transcriptomic profile [115]. Together, these studies suggest that cellular senescence can drive neurological dysfunction, including cognitive decline, and neurodegenerative pathology in both the contexts of TBI and sporadic neurodegenerative conditions. Furthermore, these studies suggest that cellular senescence may even possibly precede the emergence of pathology [146].

In our previous work, we presented widespread accumulation of DNA damage and up-regulation of DDR signaling gene expression in men with history of mTBI [135]. Here, using a case series of 38 individuals with mTBI history we show that DNA damage and markers of cellular senescence are significantly elevated in mTBI brains, and are accompanied by decreased expression of DNA repair genes, indicating inefficient DNA repair in these individuals. We suggest that concussed brains are characterized by deficient DNA repair and by DNA damage-induced cellular senescence and propose that this may be the driver of brain dysfunction associated with mTBI, and may predispose and even precede the progression to different types of neurodegenerative diseases.

## Methods

### Human brain tissue

This study has been approved by the Ethics Review Board at the Hospital for Sick Children (REB#1000059400). Cases were a collection of 38 brains donated for use in research. Cases were male, aged between 15 and 87 years old (mean age of 56.4 years), and had a history of multiple mTBIs. Nearly all of these individuals were involved in contact sports such as football, hockey, rugby, boxing, and extreme sports. Informed consent for study participation and brain autopsy was given either by the participant prior to death or by the participant’s next of kin. Controls were age-matched healthy individuals with no history of mTBI (*n* = 5) and AD cases with no history of mTBI (*n* = 5).

### Immunohistochemistry

Brains were fixed in formalin and sampled according to the National Institute on Aging Association (NIA-AA) guidelines for the neuropathological assessment of Alzheimer’s disease and other neurodegenerative diseases, including CTE [100]. Brain regions sampled included cortical, subcortical, cerebellar, and brainstem areas (up to 25 different blocks). The samples were processed and embedded in paraffin. Following embedding, formalin-fixed paraffin embedded sections (FFPE) were cut into 6 micron sections and mounted on glass slides. Each section was stained with Luxol fast blue and hematoxylin and eosin (LFB/H&E), followed by a full neuropathological assessment with the following antibodies: Phospho-Tau (Ser199, Ser202) (polyclonal rabbit, #44-768G: Thermo Scientific, 1:1000), TDP-43 (polyclonal rabbit, #PA5–29949, Thermo Scientific 1:500), β-amyloid (monoclonal mouse, DAKO, M0872, 1:50), and α-synuclein (monoclonal rabbit, Thermo Scientific #701085, 1:500). Each case was diagnosed by a staff neuropathologist and a resident in neuropathology and select cases were reviewed blindly by different neuropathologist in other institutions. In addition, to assess DNA damage each section was stained with γH2AX (monoclonal mouse, 1:1000, Ser139, #05–636; Millipore), a robust marker of double strand breaks (DSBs). Sections were also stained with GFAP (polyclonal rabbit, Omnis; Dako), H3K27Me3 (polyclonal rabbit,#07449, Millipore), lamin B1 (monoclonal rabbit, Abcam, #133741), emerin (mouse monoclonal, 1:20, Leica; United Kingdom), BRG1 (rabbit monoclonal, EPNCIR111A, 1:200, abcam), Tau100 (monoclonal mouse, MN1060, Thermo, 1:200), myelin basic protein (generously stained by Dr. W. Moore, Bristish Columbia; 1:250), and neurofilament (Abcam, 1:1000). Selected sections were double-labelled with GFAP, p-tau, and red chromogen (AEC; cat#ab64252, Abcam) combined with diaminobenzidine (DAB- Vector labs)).

### Immunohistochemistry quantification

All blocks were analyzed for γH2AX in each case. Slides were scanned with an Aperio Scanscope AT2 at 40x magnification. In each slide, three regions of interest were chosen blindly from the following areas: the ependymal lining, subependymal areas, cortical grey matter, subpial areas, and white matter. In each region the number of γH2AX-positive ependymal cells, astrocytes, oligodendrocytes, and neurons were manually counted by morphological identification and divided by the total number of each cell type in that region to create a positive cell density scores. The mean positive cell density score for each cell type was then calculated for each case. Therefore, guided by preliminary examination, we noted a consistent pattern of distribution and placed cases into three stages of positivity based on the distribution of γH2AX. Stage one was defined as having γH2AX limited to the ependymal lining of the ventricle. Stage two was defined as having γH2AX in the ependymal lining and sub-ependymal region, subpial astrocytes, as well as γH2AX-positive glia in the grey matter (peri-neuronal satellite cells). Stage three was defined has having γH2AX in the ependymal lining, γH2AX-positive astrocytes as described in stage two, and γH2AX-positive oligodendrocytes in the white matter.

### NanoString gene expression assay

Of the 38 donated brains with a past history of multiple mTBI, 11 cases had sufficiently high quality RNA for analysis of gene expression using NanoString nCounter Technology. These were compared to control brains with no history of trauma. For this study, a custom panel of genes was created consisting of 169 genes involved in the DDR and cellular senescence, including SASP factors (Additional file 1). Seven housekeeping genes were used for normalization: AARS, CYC1, GUSB, HPRT1, RPL13, TBP, and UBED2D2. Shavings from FFPE blocks containing the hippocampus were used for isolation of total RNA. This was accomplished using the RNeasy FFPE Kit by Qiagen (Qiagen Inc., Toronto, ON, Canada) with no changes to the manufacturer protocol. Total RNA was quantified using the Nanodrop 2000 spectrophotometer (NanoDrop Technologies, Wilmington, DE, USA). Two hundred nanogram RNA was used from each sample for gene expression profiling, performed with the digital multiplexed NanoString nCounter analysis system (NanoString Technologies, Seattle, WA, USA). Raw data was normalized against the six housekeeping genes. Normalized data was then analyzed and visualized using nSolver software (NanoString technologies). The log2 fold change or log2 RNA count number was calculated using normalized RNA count numbers. Statistical significance between cases and the control was determined using an unpaired student’s t-test with significance set at *p* ≤ 0.05.

## Results

### Cohort demographics and clinical presentation

Cases were between the ages of 15 and 87, with a mean age of 56.4 years old. With the exception of two cases who experienced mTBI unrelated to sport, all cases were exposed to mTBI through their involvement with contact sports including football, hockey, rugby, and boxing. These individuals, who experienced mTBI through sports-related injury, experienced chronic exposure to mTBI (length of exposure greater than 5 years).

In 35/38 (92.1%) cases with a history of mTBI, the individual suffered from either neurobehavioral and/or psychiatric symptoms (including depression, anxiety, suicide, and erratic behavior), or cognitive dysfunction and/or dementia. Two cases (5.3%) presented with motor dysfunction which was attributed to diagnosis of PD or ALS. The most common clinical characteristic of the individuals in this cohort was the presence of a mood disorder, comprising 42.1% of all cases. Non-AD control cases with no history of head trauma did not present with neurobehavioral, psychiatric, or cognitive symptoms.

### Neuropathological assessment

Each case underwent brain autopsy and a full neuropathological assessment. In this cohort, 8 (21%) cases did not have any evidence of neuropathological changes and were not given a diagnosis, despite presenting with pre-mortem neurobehavioral symptoms, leading in some cases to suicide. In 20 (53%) cases, substantial neuropathological changes were found resulting in diagnosis of a neurodegenerative disease. Included in these diagnoses were AD (40%), FTD (15%), ALS (10%), PD (5%) and non-specific tauopathy (30%). Lastly, 10 (26%) cases presented with focal, scarce perivascular p-tau lesions in the frontal cortex consistent with reported early stages of CTE [100]. Neuropathological data is being published in detail in a different publication.

### DNA damage assessment and distribution

**Fig. 1:**
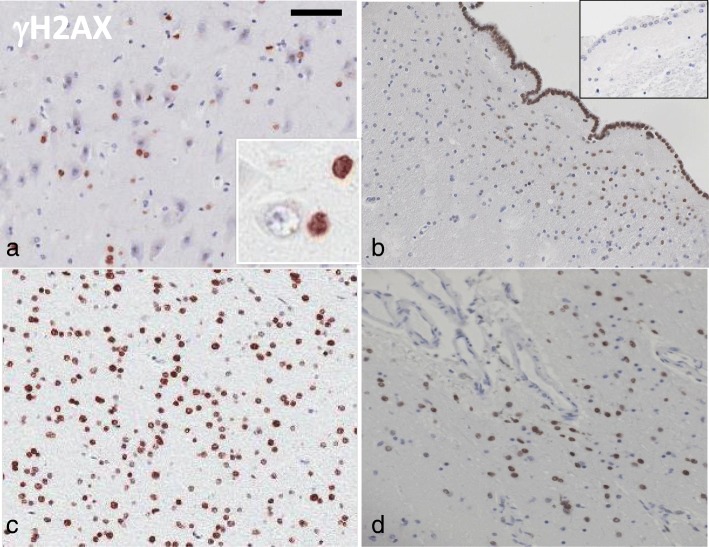
γH2AX reactivity in various cell types in cases with mTBI history. DNA damage is evident in peri-neuronal glial cells (**a**), ependymal and subependymal cells (**b**), oligodendrocytes of the white matter (**c**), and sub-pial astrocytes (**d**). In **a**, the inset shows a high power view of γH2AX-positive peri-neuronal glial cells. In **b**, the inset shows a healthy control case with no γH2AX reactivity in the ependymal lining for comparison. Scale bar represents 120 μm

**Fig. 2:**
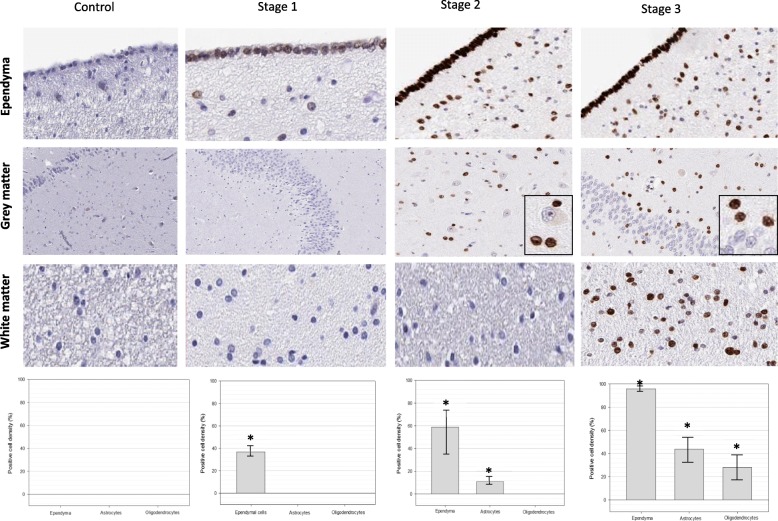
Three stages of γH2AX reactivity in cases. In stage 1, DNA damage is evident in the ependyma but not in the grey or white matter. In stage 2, DNA damage is evident in the ependymal and subependymal cells, as well as sub-pial astrocytes (not shown) and peri-neuronal glial cells in the grey matter. In stage 3 DNA damage is evident in the ependymal and subependymal cells, peri-neuronal glial cells in the grey matter, and oligodendrocytes in the white matter. Healthy, normal controls do not have any evidence of γH2AX reactivity in the ependymal, grey matter, or white matter. Stage 1 had a mean positive cell density score of 37% in ependymal cells (SE = 7.3), and 0% in astrocytes and oligodendrocytes. Stage 2 had a mean positive cell density score of 59% (SE = 19.4) in ependymal cells, 11% (SE = 5.7) in astrocytes, and 0% in oligodendrocytes. Stage 3 had a mean positive cell density score of 96% (SE = 2.7) in ependymal cells, 44% (SE = 10.9) in astrocytes, and 28% (SE = 10.2) in oligodendrocytes. Using the Kruskal-Wallis one way analysis of variance, statistically significant (*p* < 0.001) differences were observed between stages 1, 2 and 3 in the positive cell density scores in ependymal cells, astrocytes, and oligodendrocytes, marked with asterisks (*)

In 28/38 (73.7%) brains with history of mTBI, DNA damage was evident in various glial cells by immunohistochemistry with γH2AX (Fig. 1). DNA damage was seen in peri-neuronal glial cells (Fig. 1a), ependymal and subependymal cells (Fig. 1b), oligodendrocytes in the white matter (Fig. 1c), and sub-pial astrocytes (Fig. 1d). DNA damage was not seen in neurons, and spared endothelial cells. Microglial cells were not assessed in this paper. This reactivity was not seen in control cases with no mTBI history. The pattern and distribution of γH2AX staining was consistent in all cases, and was quantified and subsequently stratified into three “stages” representing the extent and distribution of DNA damage (Fig. 2). Fourteen (50%) cases were considered stage one, which presented with DNA damage throughout the ependymal lining only (Fig. 2). Five (17.9%) cases were considered stage two, which presented with DNA damage throughout the ependymal lining, and additionally in astrocytes in sub-ventricular (Fig. 2) and sub-pial areas, and peri-neuronal glial cells. Nine (32.1%) cases were considered stage three, which presented with DNA damage in the ependymal lining, astrocytes in grey matter (peri-neuronal satellite cells), sub-pial region, and surrounding cortical tissue, and additionally in oligodendrocytes of the white matter. Ten (26.3%) did not show any DNA damage as shown by γH2AX immunohistochemistry. The mean positive cell density for each cell type was calculated from three blindly chosen regions of interest from the ependymal lining, subependymal areas, cortical grey matter, subpial areas and subcortical white matter - revealing progressive increases in number of positive cells with each stage (histograms in Fig. 2)*.* Stage one cases had a mean positive cell density of 46% for ependymal cells, and no positive astrocytes or oligodendrocytes. Stage two cases had a mean positive cell density of 74% for ependymal cells, 12% for astrocytes, and no positive oligodendrocytes. Stage three cases had a mean positive cell density of 96% for ependymal cells, 51% for astrocytes, and 34% for oligodendrocytes. No γH2AX reactivity was seen in neurons. Statistically significant differences (*p* < 0.001) were detected between stages one, two, and three in the positive cell density scores for ependymal cells, astrocytes, and oligodendrocytes.

**Fig. 3:**
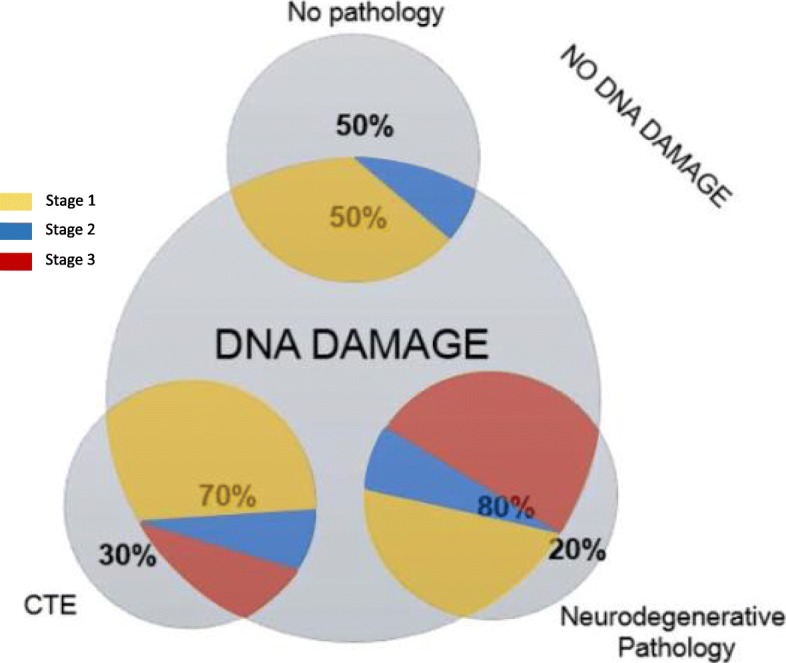
Proportion of different neuropathological changes found in 38 individuals with history of mTBI. Within the “no neuropathology” group, “CTE” group, and “neurodegenerative pathology” group, DNA damage was evident in 50, 70, and 80% of cases, respectively. Furthermore, when cases with DNA damage are stratified based on their stage of yH2AX distribution, the ‘no pathology” group presented with 75% stage 1, and 25% stage 2; the “CTE” group presented with 66.6% stage 1, 16.6% stage 2, and 16.6% stage 3; and the ‘neurodegenerative pathology” group presented with 35.3% stage 1, 17.6% stage 2, and 47.1% stage 3

When assessing γH2AX reactivity in the context of neuropathological assessment, we found that all three categories of pathological findings (no neuropathology, CTE, and neurodegenerative pathology) displayed some degree of DNA damage (Fig. 3). In the no pathology group (*n* = 8), 50% of cases presented with DNA damage. Within this group, 75% were stage one and 25% were stage two. In 70% of CTE cases (*n* = 10) DNA damage was evident, and within this 66.6% were stage one, 16.6% were stage two, and 16.6% were stage three. Lastly, in the neurodegenerative pathology group (*n* = 20), DNA damage was found in 80% of cases, with 35% being stage one, 17% stage two, and 47% stage three. Controls without any history of mTBI or neuropathologically proven neurodegenerative diseases did not show any DNA damage.

### DNA damage response pathways are activated in mTBI brains

**Fig. 4:**
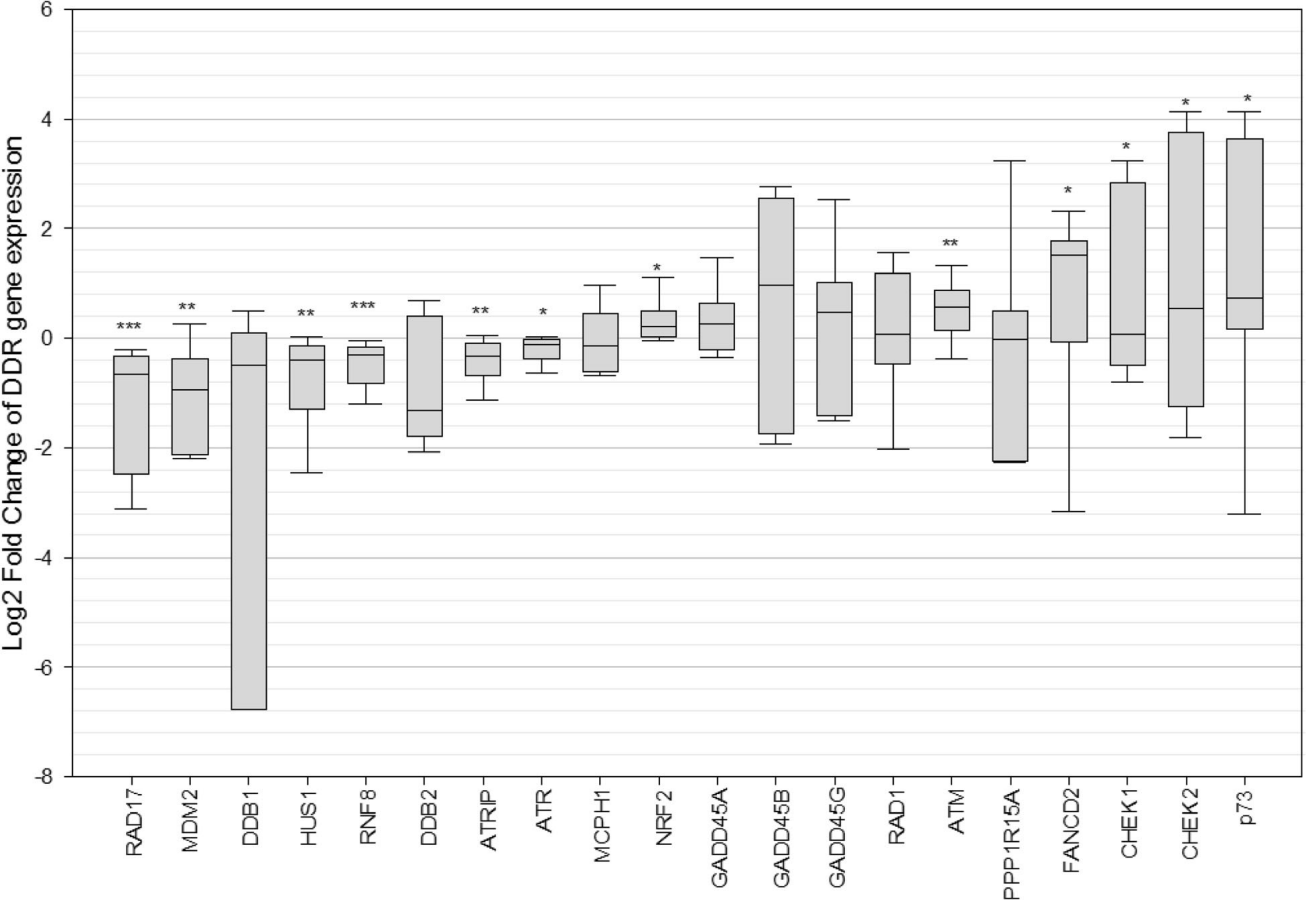
Expression of genes involved in the DNA damage response (DDR), shown as log2 fold change in cases compared to controls. Genes which are down-regulated are involved in genomic stability in response to stress (RAD17, HUS1), proteins which are degraded upon DNA damage (MDM2), proteins which respond to UV damage and single-stranded breaks (DDB1, DDB2, ATRIP, ATR), and genes whose inhibition lead to DDR-mediated cell-cycle arrest and repair (RNF8). Genes which are up-regulated are all involved in DDR signalling in response to DNA damage. Statistical significance was determined using a student t-test with significance at *p* ≤ 0.05

Expression of 32 genes involved in the DDR were analyzed. In brains with mTBI history six genes (NRF2 (*p* = 0.04), ATM (*p* = 0.01), FANCD2 (*p* = 0.01), CHEK1 (*p* = 0.04), CHEK2 (*p* = 0.05), and p73 (*p* = 0.02)) involved in the response to DNA damage were significantly upregulated when compared to controls (Fig. 4). Respectively, these genes function as antioxidants (NRF2) [117], serine/threonine kinases which phosphorylate H2AX in response to DSBs (ATM) [13], a protein ubiquitinated in response to DNA damage to localize with BRCA1 (FANCD2) [116], two checkpoint kinases activated in response to DNA damage to arrest the cell cycle (CHEK1 and CHEK2) [141], and a tumor suppressor gene which are involved in the cellular response to stress (p73) [34]. Six additional genes, also involved in the DDR and known to be upregulated following DNA damage, were upregulated in mTBI brains although these were not statistically significant. These included MCPH1, GADD45A, GADD45B, and GADD45G, RAD1, PPP1R15A (Fig. 4).

Cases also displayed significant decreased expression of two genes for which loss is associated with loss of genomic integrity (Fig. 4). These include MDM2 (*p* = 0.05) which is degraded upon DNA damage [163], RNF8 (*p* = 0.0009) whose inhibition is associated with defects in DNA repair [151]. Interestingly, cases showed decreased expression of genes involved in the DDR specifically to single-stranded DNA breaks, although not all of these were statistically significant. These included DDB1 (*p* = 0.05), DDB2 (*p* = 0.24), ATRIP (*p* = 0.01), and ATR (*p* = 0.02). Additionally, two genes encoding checkpoint proteins, RAD17 (*p* = 0.0003) and HUS1 (*p* = 0.003) were significantly downregulated in cases (Fig. 4). Depletion [39] and inactivation [165] of these genes, respectively, has been linked to genomic instability in response to cellular stress, such as DNA damage.

### Altered expression of DNA repair genes in mTBI brains

**Fig. 5:**
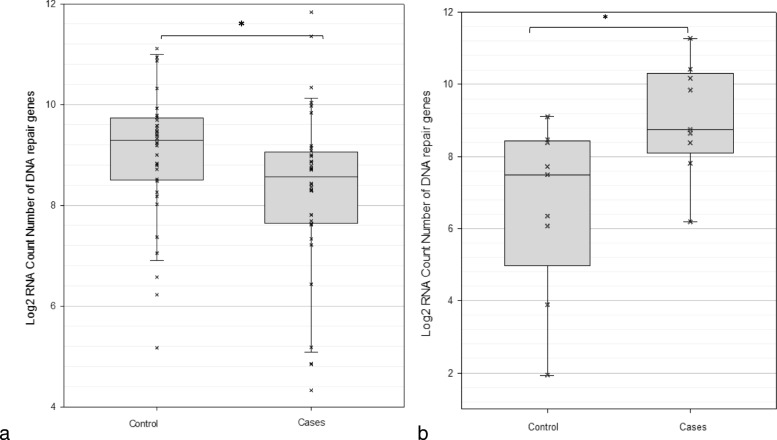
Altered expression of DNA repair genes in mTBI brains compared to controls, expressed as log2 RNA count number. In **a**, significantly down-regulated DNA repair genes (*n* = 37) are: MSH4 (*p* = 0.0001), FANCA (*p* = 0.0001), POLD1 (*p* = 0.0002), NBN (*p* = 0.001), RAD23B (*p* = 0.0001), APEX1 (*p* = 0.004), POLB (*p* = 0.003), MBD4 (*p* = 0.007), RAD9A (*p* = 0.005), ERCC2 (*p* = 0.01), XPA (*p* = 0.02), ERCC8 (*p* = 0.02), XAB2 (*p* = 0.02), CETN2 (*p* = 0.02), DMC1 (*p* = 0.004), XRCC6 (*p* = 0.002), PMS1 (*p* = 0.03), XRCC3 (*p* = 0.03), H2AFX (*p* = 0.004), BMI1 (*p* = 0.04) PMS2 (*p* = 0.003), RAD21 (*p* = 0.02), MSH3 (*p* = 0.02), MLH3 (*p* = 0.04), ERCC3 (*p* = 0.01), RAD23A (*p* = 0.01), RAD51C (*p* = 0.0001), NEIL2 (*p* = 0.02), MDC1 (*p* = 0.007), RNF168 (*p* = 0.02), MSH2 (*p* = 0.0001), XRCC5 (*p* = 0.003), NTHL1 (*p* = 0.02), RPA2 (*p* = 0.002), NEIL1 (*p* = 0.006), RAD50 (*p* = 0.04). In **b**, significantly up-regulated DNA repair genes (*n* = 9) are: RAD51B (*p* = 0.03), RAD52 (*p* = 0.05), RAD18 (*p* = 0.05), RAD54L (*p* = 0.05), MSH5 (*p* = 0.03), BRCA2 (*p* = 0.01), MAG1 (*p* = 0.05), TDG (*p* = 0.04), NEIL3 (*p* = 0.03)

Expression levels of 85 genes encoding DNA repair proteins were analyzed and 45 were significantly altered in their expression levels. DNA repair genes displayed a general trend towards decreased expression with 36 genes significantly down-regulated (Fig. 5a) and 9 genes significantly up-regulated (Fig. 5b) in mTBI brains compared to a control (*p* ≤ 0.05). The remaining 40 genes did not show any statistically significant changes in gene expression between cases and controls. Notably, some DNA repair genes which were up-regulated in cases have more than one functional role which should not be overlooked. For example, RAD51B (*p* = 0.02) has been reported to cause cell-cycle delay when overexpressed [54]. Similarly, RAD18 (*p* = 0.03) has been reported to activate cell-cycle checkpoints via DNA damage signaling [156], again consistent with the induction of cell-cycle arrest during cellular senescence.

### Activation of cellular senescence pathways in mTBI brains

**Fig. 6:**
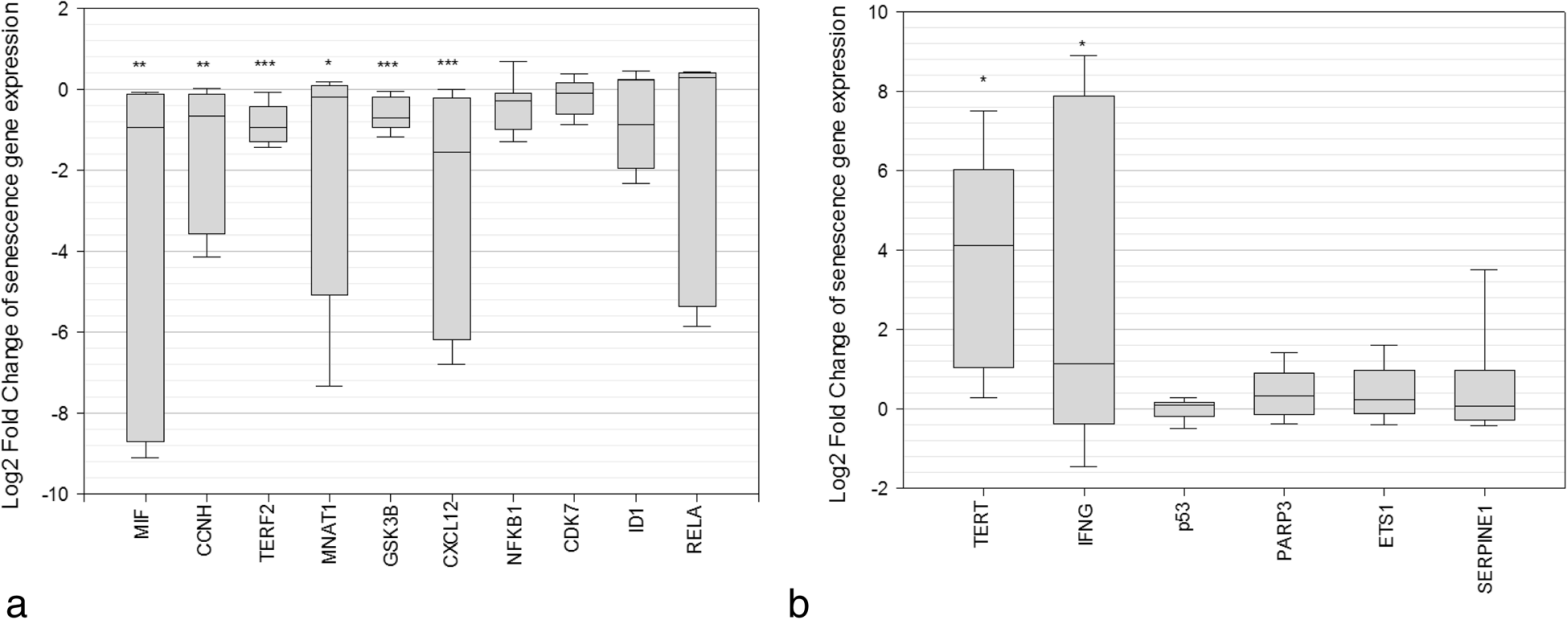
Altered expression of genes involved in the senescence phenotype in cases compared to controls, expressed as log2 fold change. Down-regulated genes (**a**) have roles in protecting cells against the senescent phenotype in mTBI cases. Upregulated genes (**b**) have roles in driving the senescent phenotype in mTBI cases, expressed as log2 fold change in RNA count number compared to a control

Seventeen genes were analyzed for their involvement in the senescent phenotype. Five genes with general functions in protecting gene integrity and delaying senescence were significantly down-regulated in mTBI brains compared to controls (Fig. 6a). These include MIF (*p* = 0.003), CCNH (*p* = 0.004), TERF2 (*p* = 0.001), MNAT1 (*p* = 0.04), and GSK3B (*p* = 0.0003). Defects in these genes have been associated with senescence (MIF) [166], dysregulation of cell-cycle kinases (CCNH) [162], loss of telomere integrity (TERF2) [8], loss of CDK kinase activation (MNAT1) [172], and induction of senescence (GSK3B) [76] respectively. ID1, RELA, NFKB1, and CDK7 were also down-regulated, consistent with reports on senescence, however these were not statistically significant. One chemokine, for which decline has been associated with cognitive decline in AD [123], CXCL12, was significantly down-regulated (*p* = 0.0006) in concussed brains.

Two genes associated with senescence were significantly up-regulated in mTBI brains (Fig. 6b). TERT, which is up-regulated in response to shortened telomeres [96], was significantly over-expressed (*p* = 0.04) in mTBI brains. Furthermore IFNG, a soluble cytokine which induces senescence through p53 signaling [75], was significantly over-expressed (*p* = 0.04) in mTBI brains. Four other genes which are positively associated with senescence were slightly upregulated, (p53, PARP3, ETS1, and SERPINE1), although these were not statistically significant (Fig. 6b).

### Increased expression of pro-inflammatory SASP factors in mTBI brains

**Fig. 7:**
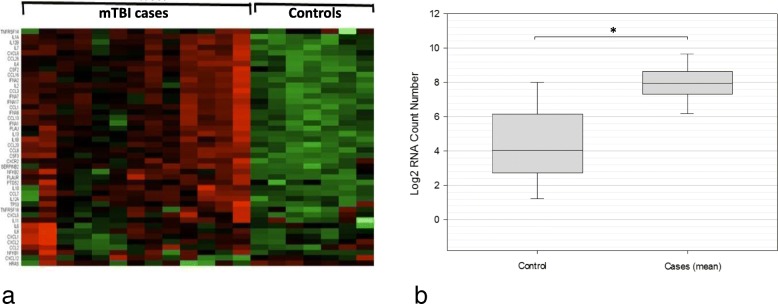
Upregulation of pro-inflammatory SASP factors in cases compared to controls. In a heatmap representing gene expression **a**, cases display high expression (indicated in red) of pro-inflammatory SASP factors compared to various controls which show low expression (indicated in green). Furthermore, representation of log2 RNA count number of various SASP genes (**b**) shows significant up-regulation of these genes in cases compared to controls. The genes shown here are: CCL1*, CCL11*, CCL13*, CCL16*, CCL2, CCL20*, CCL25*, CCL26*, CCL3*, CCL4***, CCL8***, CXCL1**, CXCL11*, CXCL2, CXCL3, CXCL5*, CXCL6*, CXCL8*, CXCR2*, IL11, IL12A, IL12B*, IL13*, IL15*, IL1A*, IL1B*, IL2*, IL4*, IL6*, IL7*, Il6R, NFATC1***, NFATC2**, and NOX4*. Statistical significance was determined using an unpaired student’s t-test with significance denoted at *p* ≤ 0.05

Compared to controls, brains with history of head trauma showed significant increased expression of several pro-inflammatory chemokines, cytokines, interleukins, and receptors (Fig. 7a and b). Of these 35 genes, 28 were detectable in our tissue above background levels: CCL1 (*p* = 0.04), CCL11 (*p* = 0.02), CCL13 (*p* = 0.02), CCL16 (*p* = 0.03), CCL20 (*p* = 0.04), CCL25 (*p* = 0.03), CCL26 (*p* = 0.01), CCL3 (*p* = 0.03), CCL4 (*p* = 0.0001), CCL8 (*p* = 0.0007), CXCL1 (*p* = 0.005), CXCL11 (*p* = 0.01), CXCL5 (*p* = 0.02), CXCL6 (*p* = 0.03), CXCL8 (*p* = 0.02), CXCR2 (*p* = 0.02), IL12B (*p* = 0.03), IL13 (*p* = 0.02), IL15 (*p* = 0.0142), IL1A (0.03), IL1B (*p* = 0.02), IL2 (*p* = 0.03), IL4 (0.03), IL6 (0.04), IL7 (0.02), and IL6R (*p* = 0.0004). Additionally, mTBI brains showed significantly increased expression of two other pro-inflammatory genes, NFATC2 (*p* = 0.007), and NOX4 (0.04). These genes encode a transcription factor which regulates the immune response [7] and an NADPH oxidase which produces ROS and contributes to genomic instability and DNA damage [106], respectively.

### Loss of nuclear proteins as markers of cellular senescence

**Fig. 8:**
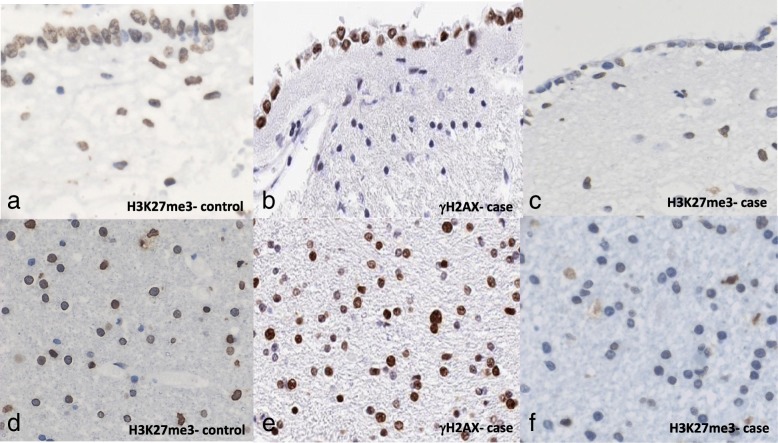
H3K27Me3 is normally expressed in the ependymal lining (**a**) and in oligodendrocytes of the white matter (**d**) of a healthy control, but in mTBI cases with evidence of DNA damage (**b, e**) H3K27Me3 expression is lost in the ependymal lining (**c**) and oligodendrocytes (**f**)

**Fig. 9:**
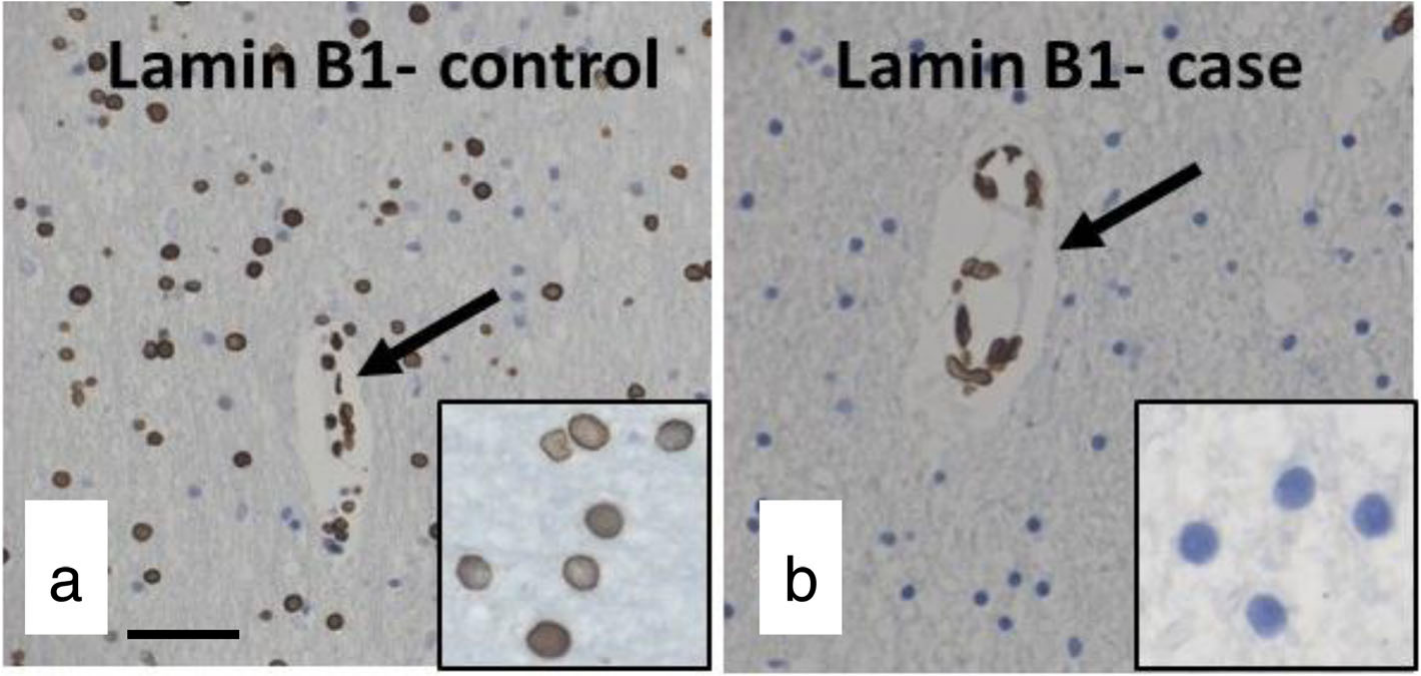
Lamin B1 is normally expressed on the nuclear membrane within the white matter of a healthy control (**a**) but is lost in a case with mTBI history (**b**). Noticeably, lamin B1 was conserved in endothelial cells of cases (arrow in **b**) comparable to expression in controls (arrow in **a**). High power views of panels **a** and **b** are shown as insets. Scale bar represents 50um

Cases with history of mTBI had decreased immunohistochemical expression of two nuclear proteins, loss of which are considered markers of cellular senescence. First, the epigenetically modified histone H3K27Me3 is normally expressed in controls (Fig. 8a and d), but is lost in areas with γH2AX positivity (Fig. 8b and e) in mTBI brains (Fig. 8c and f) shown here in the ependymal lining and white matter (but also affecting other glial cell types). This histone typically forms heterochromatic regions for transcriptional repression [64], and is normally associated with repair of DSBs through transcriptional regulation of the p21 pathway [170]. However loss of H3K27Me3 in the context of DNA damage has been reported to induce cellular senescence through activation of p16 and p21 [66]. The second protein lost in mTBI brains compared to controls was lamin B1. Indeed, lamin B1 was normally expressed in control brains (Fig. 9a) but reduced in expression in our cases (Fig. 9b). This nuclear envelope protein typically tethers chromatin to the nuclear membrane to silence gene expression [15], and its loss results in widespread chromatin rearrangement and significant changes in gene expression reflective of senescence [138]. Indeed, loss of lamin B1 is considered a biomarker of senescence [40] and has been reported to underlie the progression of several tauopathies and neurodegenerative diseases [61].

### Morphological changes consistent with cellular senescence in mTBI brains

**Fig. 10:**
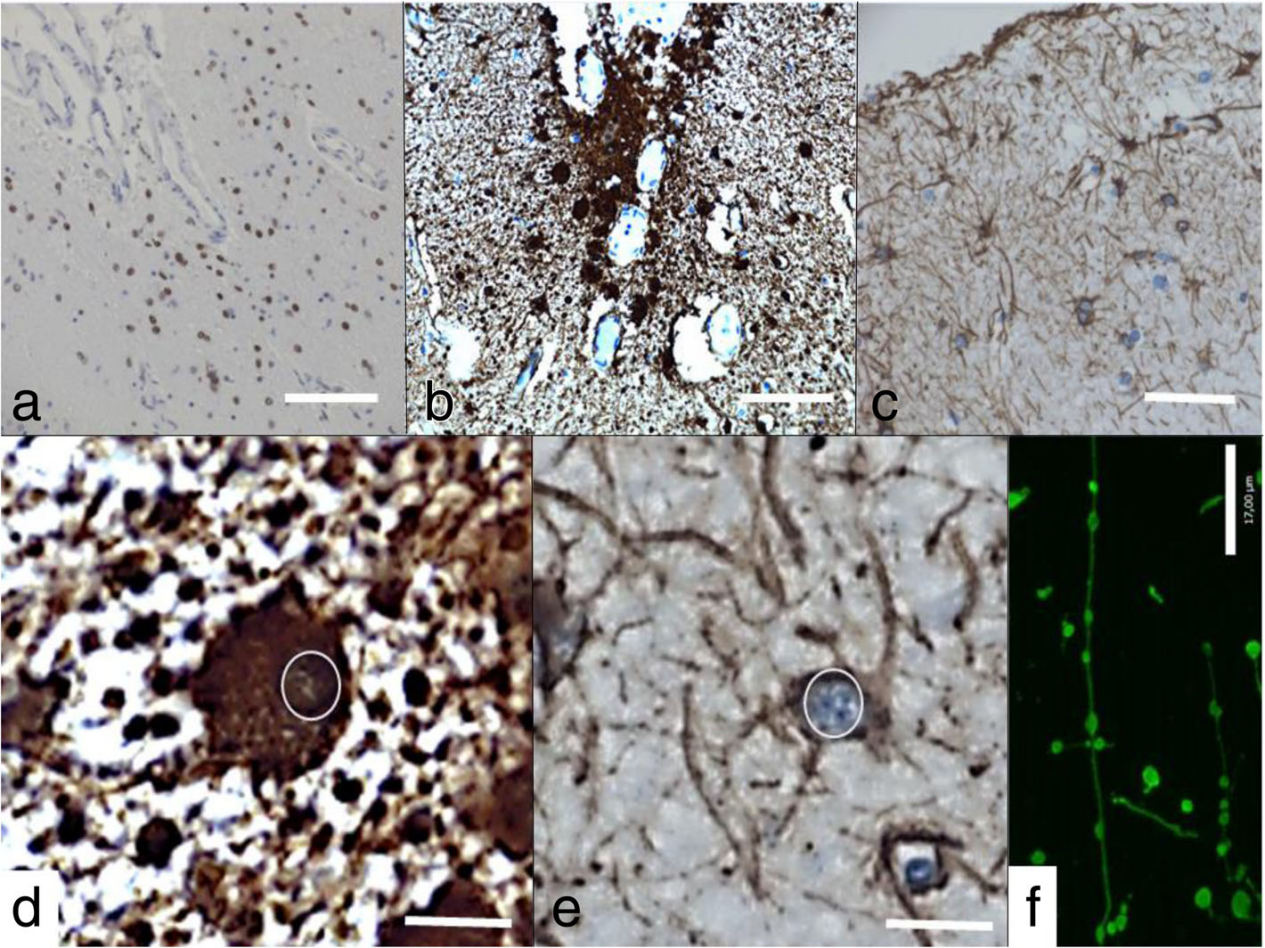
GFAP-positive immunostaining of sub-pial astrocytes in the same region as yH2AX (**a**) shows abnormal ballooning of the cell body of astrocytes in the sub-pial area (**b** and **d**) when compared to a comparable section from healthy control (**c** and **e**). The cell body in a case (**d**) is abnormally swollen compared to that of a healthy control (**e**). The circles in **d** and **e** show comparable nuclear sizes in case and control but swollen cell body of astrocyte is evident in case. GFAP immunofluorescence (**f**) reveals beading of astrocytic processes in individuals with mTBI history. Scale bar represents 100 μm in (**a** and **b**), 40 μm in (**c**), 10 μm in (**d** and **e**) and 17 μm in (**f**)

**Fig. 11:**
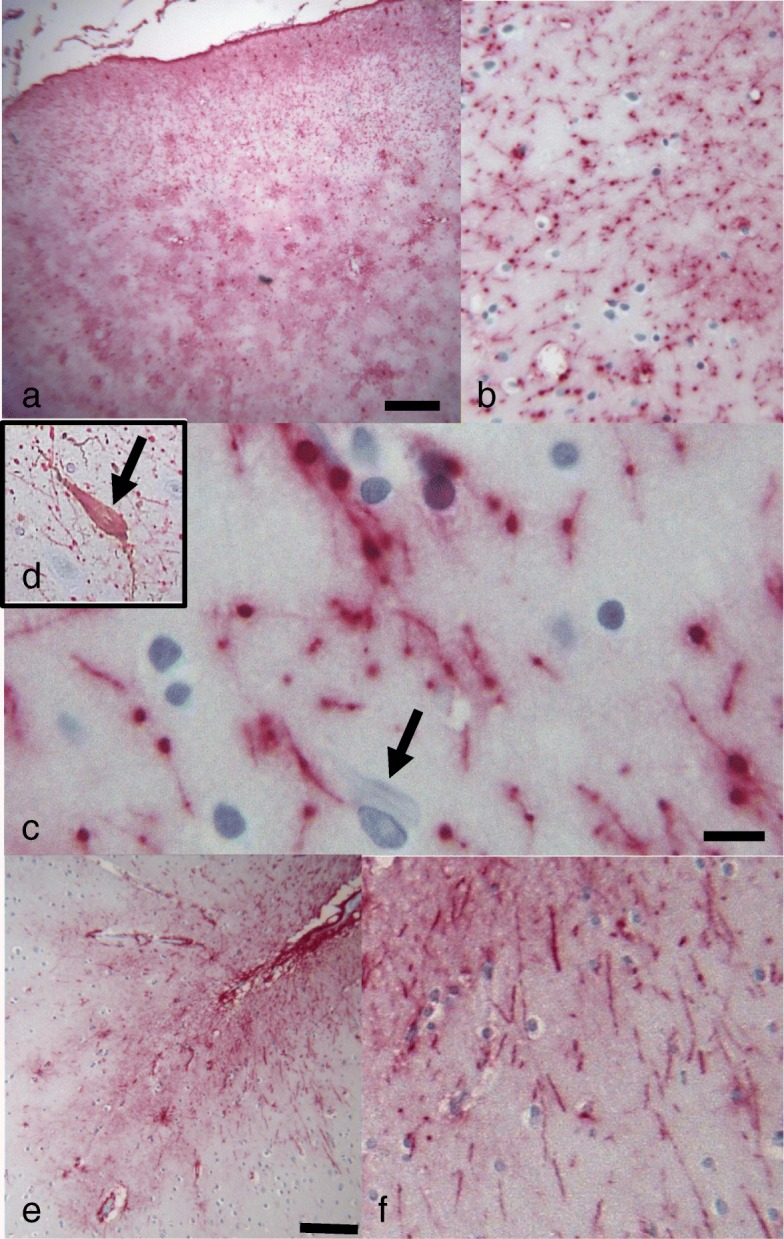
**a** and **b** Photomicrographs showing sub-pial positive GFAP immunostaining in an TBI case. Sub-pial astrocytes show beaded processes (**c** and **d**). Neurons with neurofibribrillary tangle (arrow) and as shown by double labeling for GFAP (red chromogen) and p-tau (AT8- brown chromogen) in inset are noted in the same area. Photomicrographs showing sub-pial GFAP positive astrocytes in the subpial region of a control case, illustrate tha absence of beading. (**e** and **f**). Scale bar in **a** represents 500 μm in (**a**) and 150um in (**b**), 30um in (**c**) and 60um in (**d**), 300um in (**e**) and 120um in (**f**)

In support of our findings indicating increased expression of senescence driving genes and SASP, we studied astrocytes which were positive for markers of DNA damage. Cellular senescence in astrocytes is marked by significant swelling and enlargement of the cell bodies, and beading of cellular processes [21]. These changes are thought to reflect increased levels of transcription and secretion of pro-inflammatory SASP factors [132]. In our cohort, astrocytes which were γH2AX positive (Fig. 10a) in individuals with history of mTBI showed significant changes consistent with morphological features associated with cellular senescence (Fig. 10). Indeed, GFAP reactivity in serial sections of mTBI revealed that astrocytes with DNA damage were enlarged, presenting with swollen cytoplasm (Fig. 10b and d) compared to healthy, normal astrocytes (Fig. 10c and e). Immunofluorescence and immunohistochemistry for GFAP revealed substantial axonal beading of cellular processes in damaged astrocytes (Figs. 10f and 11a-c). The processes of subpial astrocytes were abnormally beaded in mTBI cases with evidence of DNA damage (Fig. 11a-c). Tau-positive neurofibrillary tangles could be found in brain areas with abnormal astrocytes (Fig. 11c and d). In contrast, sub-pial GFAP-positive processes were normal in control cases with no evidence of DNA damage (Fig. 11e and f).

### Neuronal changes in cases with DNA damage

**Fig. 12:**
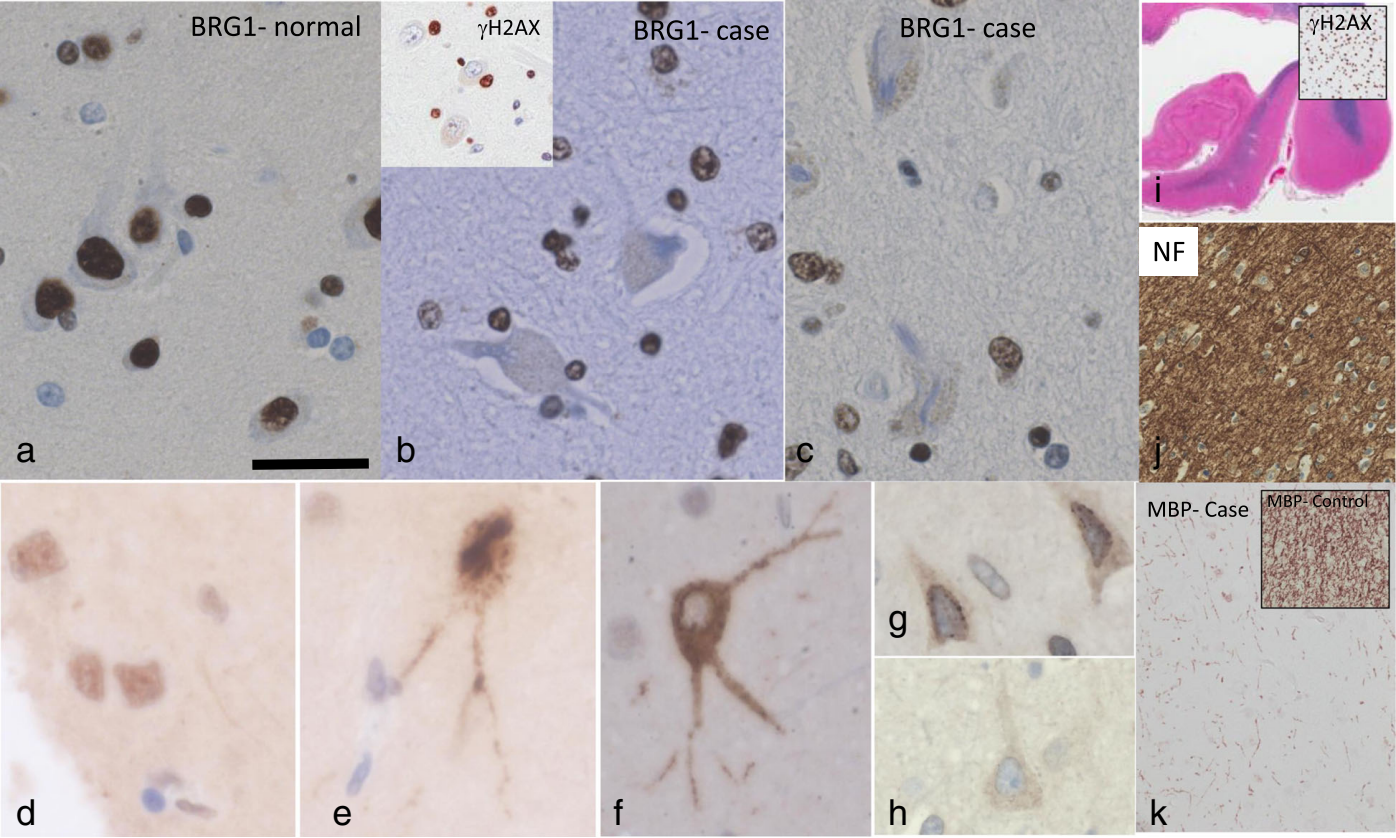
Neuronal changes in mTBI cases. BRG1 is normally expressed in neurons and glial cells in a healthy control (**a**), but its expression is lost in neurons in a cases with increased glial γH2AX expression (**b-c**). Translocation of intranuclear tau from the nucleus to the cyroplasm was evident in mTBI cases (**e-f**) and not seen in controls (**d**). In addition, emerin expression on the nuclear membrane of neurons was normal in controls (**g**) but lost in mTBI cases (**h**). Lastly, mTBI cases with DNA damage displayed white matter pallor (**i**), as well as intact neurofilament protein expression (**j**) but loss of myelin basic protein expression (**k**) compared to controls (inset in **k**). Scale bar represents 40 μm in (**a-h**); 50 μm in (**d**)

In order to investigate neuronal changes in cases with evidence of glial cell senescence, we stained for proteins with implications in genome integrity, nuclear membrane structure, and axonal and myelin composition. Compared to normal controls (Fig. 12a), mTBI cases with evidence of DNA damage in glial cells (Fig. 12b inset) showed loss of Brahma-related gene-1 (BRG1/SMARCA4) nuclear expression selectively in neurons, but was retained in glial cells (Fig. 12b). In neurons, BRG1 is a transcription factor which is critical for neuronal development, gliogenesis, and normal gene expression [refs], and for which loss has been associated with neurodegeneration and neuronal cell loss [31]. In addition, cases with evidence of DNA damage in glial cells showed translocation of intranuclear tau to the cytoplasm (Fig. 12d-f). Although tau protein is most commonly associated with its microtubule organizing properties in the axonal cell compartments and its pathogenic hyperphosphorylation in AD, a distinct isoform of tau has recently been found to localize in the nucleus and function to tether chromatin for nucleic acid stabilization [refs]. Furthermore, translocation of intranuclear tau to the cytoplasm has recently been associated with loss of DNA integrity and neurodegenerative disease [57, 158]. Cases with DNA damage in glial cells also presented with loss of the nuclear envelop structural protein emerin (Fig. 12h) compared to controls (Fig. 12g). Emerin is a structural integral protein which acts to stabilize chromatin and regulate gene expression [108]. We also observed significant white matter changes in mTBI cases with glial cell DNA damage compared to controls. First, white matter pallor was visible in cases with evidence of DNA damage in oligodendroglial cells (Fig. 12i). Using immunohistochemistry, we found intact neurofilament protein in this region (Fig. 12j), but significant loss of myelin basic protein (MBP) expression (Fig. 12k). Myelin basic protein is a key structural protein for myelin, comprising approximately 30% of all myelin protein in the central nervous system [110], and for which loss has been associated with ageing and neurodegenerative conditions [164].

### DNA damage burden and proteinopathy

**Fig. 13:**
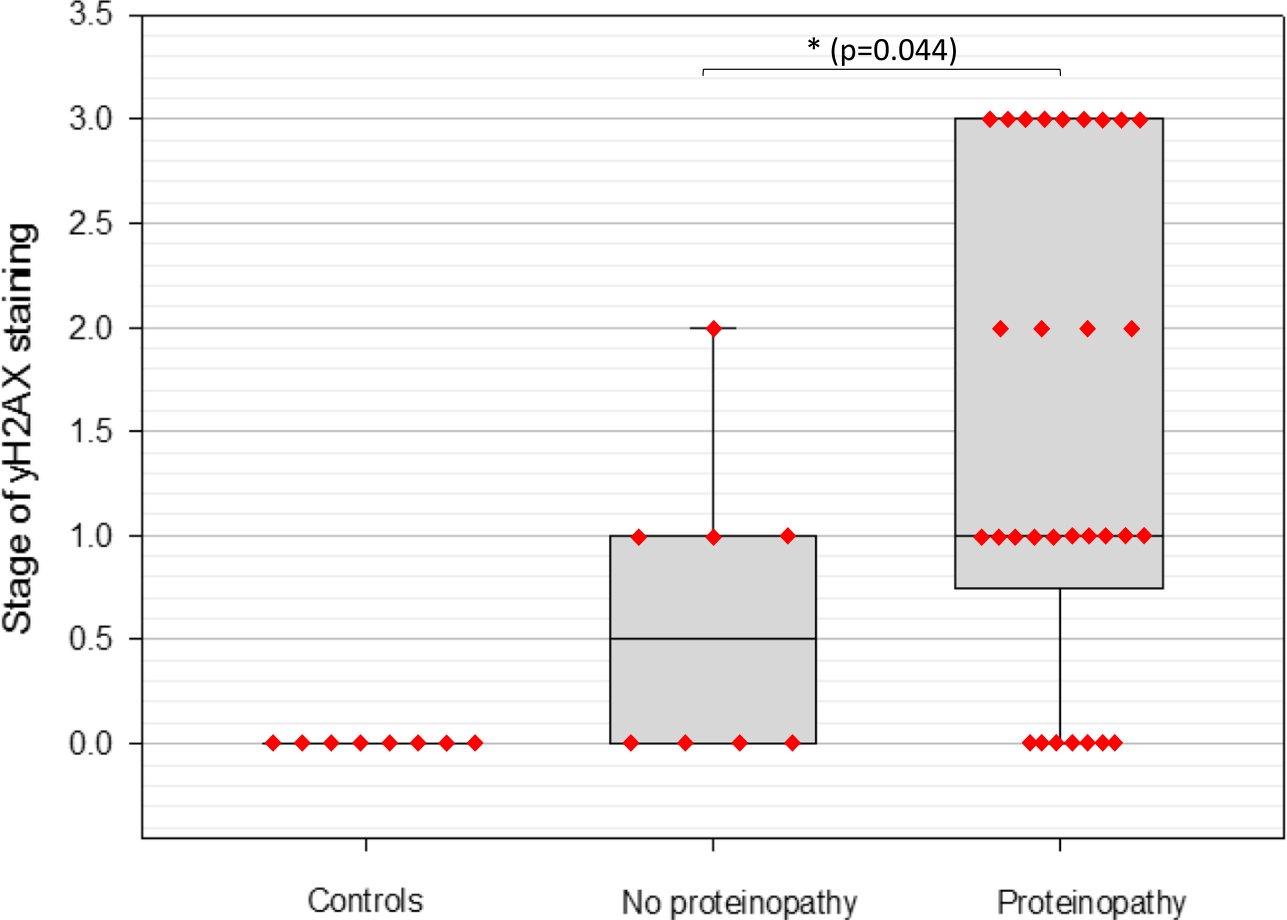
Correlation between proteinopathy and stage of yH2AX distribution in cases and controls with no mTBI history. The presence of a proteinopathy was significantly correlated with higher levels of DNA damage in brains with a history of mTBI (*p* = 0.04, Mann-Whitney Rank Sum Test). Cases with a proteinopathy presented with abnormal accumulation of at least one of the following proteins: p-tau, β-amyloid, α-synuclein, or TDP-43. Individuals with no proteinopathy did not present with abnormal accumulation of any of the listed proteins

Cases with mTBI history were stratified based on the absence or presence of proteinopathy. Using immunohistochemistry, cases were analyzed for the presence of abnormal protein depositions including hyperphosphorylated tau, β-amyloid, α-synuclein, and TDP-43. Cases which presented with any of the above toxic proteins were placed into the “proteinopathy” group (*n* = 30), whereas cases with none of the above proteins were placed into the “no proteinopathy” group (*n* = 8). Cases with a proteinopathy were, on average, older (63.28 years) than those without proteinopathy (35.62 years). The presence of a proteinopathy in individuals with mTBI history was significantly correlated with higher levels of DNA damage (*p* = 0.04, Mann-Whitney Rank Sum Test) (Fig. 13). Indeed, the proteinopathy group had a mean and median γH2AX stage of 1.55 and 1 respectively, compared to a mean and median γH2AX stage of 0.62 and 0.5 respectively in the non-proteinopathy group (Fig. 13).

**Fig. 14:**
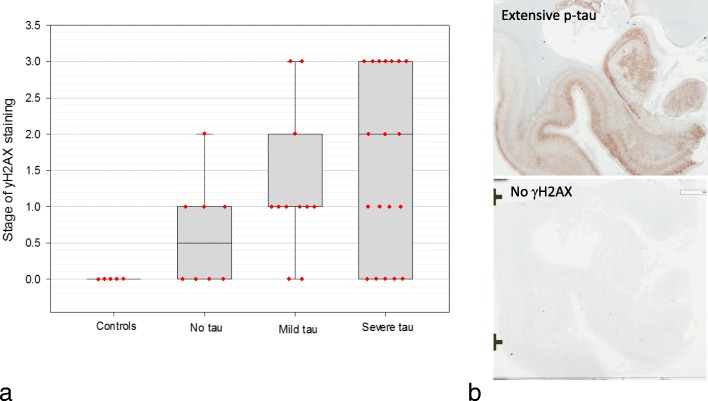
Correlation between stage of yH2AX staining and burden of tau pathology (**a**). Controls had no history of mTBI and no pathology (AD cases were excluded from controls in the figure). In the control group, no γH2AX was found. However, mTBI brains with no tau pathology presented with expression ranging from none to stage 2. The association between p-tau pathology and γH2AX expression was not statistically significant and mTBI cases with mild or severe tauopathies ranged from no no γH2AX to stage 3 γH2AX. The mean level of yH2AX staining in the no tau, mild tau, and severe tau groups were 0.6, 1.2, and 1.6, respectively, increasing progressively. In some cases of severe tauopathy γH2AX was not detected by immunohistochemistry (**b**)

Despite this association, not all cases with proteinopathy showed DNA damage. To further elaborate on a potential relationship between abnormal protein deposits and the extent and distribution of DNA damage, we specifically analyzed and correlated p-tau burden with stage of γH2AX staining. The level of tau burden was stratified into three groups: no tau, mild tau, or severe tau. The no tau group showed no p-tau reactivity throughout the brain, the mild tau group showed p-tau distribution in three or more cortical lobes in the form of isolated foci at the depths of sulci, or hippocampal tau (Braak I/II) with less than three cortical lobes affected by tau, and the severe tau group showed p-tau distribution in three or more cortical lobes with diffuse pathology and diffuse hippocampal p-tau and/or subcortical p-tau. When stratified this way, there was no statistically significant association between burden of tau pathology and stage of DNA damage (Fig. 14a). Indeed, in both mild and severe tauopathies, the DNA damage ranged from stage 0 to stage three, with some cases accumulating extensive tau but presenting with no DNA damage (Fig. 14b). Furthermore, numerous cases with no tau deposits did show DNA damage, but generally remained within the lower stages of distribution, primarily confined to the ependymal lining, suggesting that DNA damage precedes p-tau accumulation. Importantly, controls with no history of mTBI as well as no tau burden did not have any evidence of DNA damage (stage 0) (Fig. 14a). In contrast, some brains with history of mTBI while still having no tau deposits displayed stage one or stage two DNA damage (Fig. 14a). In general, there was an increased burden of DNA damage (i.e. higher stages) with increased levels of abnormal protein deposits (Fig. 14a). However, in some cases with severe tauopathy, γH2AX was not detectable by immunohistochemistry. These cases when analyzed individually did show markers of DDR and senescence as detected by NanoString, which may indicate the loss of γH2AX expression and not necessarily absence of DNA damage.

## Discussion

**Fig. 15:**
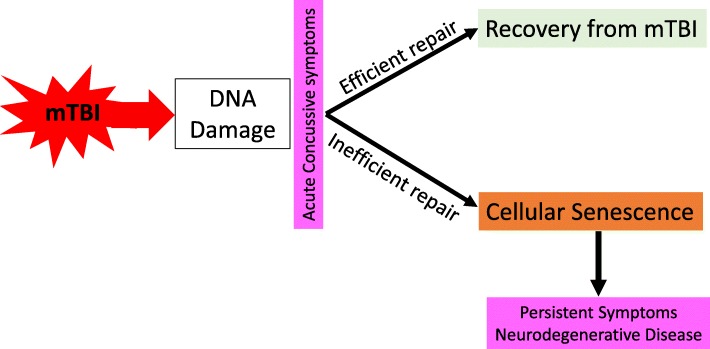
Proposed pathophysiological mechanism of mTBI-related brain dysfunction. mTBI causes the accumulation of DNA damage, which may either be efficiently repaired by DNA repair enzymes or, in some individuals who have inefficient DNA repair mechanisms, may accumulate and cause the acquisition of cellular senescence. Accumulation of senescent cells in the brain drives the persistent symptoms of mTBI and may drive the development of neurodegenerative conditions and cognitive decline later in life associated with mTBI

Involvement in contact sports and a history of multiple concussions has been shown to cause long-term effects on brain health [27, 88, 95] and many studies have credited neurodegenerative diseases as the chief driver of these long-term symptoms and pathological changes [3, 102, 144, 145]. mTBI has been linked to several different neurodegenerative diseases, and in particular CTE has been proposed as the pathological signature of concussion and the driver of symptoms [107]. However, a major gap in knowledge in our understanding of mTBI-associated brain dysfunction is the discordance between the pathology load and severity of symptoms, often in younger individuals: cases with a history of head trauma often have microscopic foci of proteinopathy, yet present with severe and diverse symptoms [134]. CTE is pathologically defined by the presence of focal perivascular p-tau in neurons and astrocytes, in the depths of cortical sulci [100]. The diagnostic criteria for CTE has no lower bound, meaning that one focus of p-tau is sufficient for a diagnosis [100]. However, CTE is described clinically by a set of non-specific but yet severe symptoms including depression, anxiety, motor dysfunction, aggression, irritability, memory loss, and suicidality [4, 68, 94]. It is very unlikely that such focal pathology is the cause of these functional deficits. In a recent study, brains of former athletes underwent clinical and neuropathological evaluation in order to establish a clinicopathological diagnosis. Although CTE-consistent pathology was found in 73% of cases, all of these presented with multiple mixed neurodegenerative pathologies, most notably AD-consistent pathology. Furthermore, the authors concluded that the clinical significance of CTE-pathology remains uncertain, and that it is likely a co-morbidity rather than the cause of dementia in former athletes [83]. It is also important to note that CTE-consistent pathology has been reported in cases with no history of head trauma, further indicating that it may be a common pathology within the general population and not specific to the aftermath of mTBI [55, 69, 89, 119]. We therefore suggest that there are other molecular processes driving symptoms and perhaps driving pathology seen after mTBI. In particular, cellular senescence can cause significant effects on brain health and function by affecting various cell types and spreading its effects to surrounding tissue [48]. Indeed, cellular senescence is thought to underlie various neurological symptoms. Enhanced levels of SASP factors have been associated with late-life depression [32], accumulation of senescent cells are thought to contribute to anxiety [60], and inducing accelerated senescence in a mouse model has been shown to result in motor dysfunction and cognitive decline [5]. Cellular senescence may therefore be one of the main mechanism by which mTBI leads to the presentation of diverse, widespread, and debilitating symptoms in individuals who experience mTBI, and represents a more plausible mechanistic explanation to mTBI-induced brain dysfunction with numerous possible targets for early diagnosis, prognosis, and treatment (Fig. 15).

### mTBI brains show progressive DNA damage and senescence in glial cells

In this study, we showed that brains from individuals with history of mTBI present with both DNA damage and gene expression changes supportive of the acquisition of cellular senescence. There were different degrees of DNA damage in glial cells, and genes which drive and protect against senescence were up-regulated and down-regulated, respectively. Furthermore, genes encoding pro-inflammatory SASP factors such as interleukins (including IL1β, IL6, and IL7) and chemokines (including CCL1, CCL3, AND CCL8), were up-regulated in mTBI brains. Together, this indicates that brains with history of mTBI have entered a state of senescence. As shown by immunohistochemistry for γH2AX, senescent cells were widespread, and accumulated in a specific and consistent pattern of distribution. In some cases, damage was limited to the ependymal lining of the ventricles and in others, it was more extensive and involved more brain areas and additional cell types including astrocytes and oligodendrocytes. The consistent pattern of γH2AX distribution led us to define three stages of DNA damage/senescence as described and illustrated in the result section. It is important to note that it is unclear from this study whether our defined three stages are a reflection of disease progression, starting in the ependymal lining and spreading to other brain regions and glial cells. However, we did find a tendency for γH2AX reactivity to increase with augmented p-tau burden, suggesting a progressive relationship between DNA damage and tauopathy, which will be discussed below further.

Senescence has the potential to spread damage to surrounding tissue via paracrine signaling with SASP factors, while simultaneously using autocrine reinforcement to maintain their senescent phenotype [48]. Senescent cells also use a series of positive feedback loops in order to spread senescence to surrounding cells, causing overall tissue dysfunction [73]. Thus, a progressive increase in senescent cell burden beginning in the ependymal lining is a possibility. In particular, the consistent pattern of distribution of γH2AX in ependymal cells suggests a potential role of the cerebrospinal fluid (CSF) in spreading senescence to surrounding tissue, via the paracrine signalling. This may possibly explain the widespread nature of symptoms from mTBI despite cases often presenting with scarce and focal pathology. In a recent paper on obesity-induced senescence, Ogrodnik et al. reported accumulation of senescent glial cells in the periventricular region of the lateral ventricle, and associated this with anxiety and impaired neurogenesis [120]. Furthermore, both pharmacological and pharmacogenetic clearance of these senescent cells alleviated anxiety behavior and enhanced adult neurogenesis [74]. This study suggests that accumulation of senescent cells in a periventricular distribution can be sufficient for the acquisition of neurological symptoms. Secretion of pro-inflammatory SASP factors into the CSF by senescent ependymal cells likely plays a role in emerging symptoms. For instance, high levels of interleukin-6 and 8, two key mediators of the SASP, are found to be significantly elevated in the CSF of aged women with clinical depression [74] compared to those without depression, suggesting a role of these pro-inflammatory factors in producing psychiatric symptoms. High levels of inflammatory markers in the CSF are also associated with increased fatigue and depression, two commonly reported symptoms in mTBI [58]. In addition, high levels of SASP factors in the CSF has been suggested to play a role in increasing susceptibility to neurodegenerative disorders [47]. Indeed, neuroinflammation markers in the CSF are strongly associated with cerebral tau pathology in older adults [127]. In contrast to these findings, concentrations of β-amyloid in the CSF of AD patients has been found to not significantly associate with cognitive performance [53, 125]. Furthermore, p-tau in the CSF has been shown to not correlate to any neuropsychological changes [53], and are not associated with AD progression in APOE3-carrying AD patients [77]. Taken together, these findings suggest that the secretion of pro-inflammatory factors by ependymal senescent cells into the CSF, as described in our cohort, may be sufficient to drive the emergence of various symptoms, and may be more suitable biomarkers of mTBI-related brain dysfunction than proteinopathy. In this way, stage one γH2AX reactivity may have substantial effects on neurological functioning, despite DNA damage appearing focal and limited to the ependymal lining.

In our understanding of emerging symptoms from damage-induced cellular senescence, it is also important to consider that cases without γH2AX reactivity are not necessarily without DNA damage. In fact, it has been shown that exposure to chronic oxidative stress and deficient antioxidant responses lead to degradation of the H2AX protein by the proteasome [50]. It may therefore be possible that some individuals in our cohort have DNA damage in forms not captured by this assay, as they no longer possess any H2AX to mount a response to DSBs. This may be especially relevant in chronic cases of mTBI, in which the individual has experienced numerous mTBIs throughout a long professional sports-playing career. Several cases in our cohort presented with a severe tauopathy, yet had no evidence of DNA damage. We suspect that in these individuals H2AX has been degraded over time, due to chronic levels of oxidative stress caused by cellular senescence. Indeed, we suggest that these individuals acquired cellular senescence during their playing career from multiple mTBIs, and that this chronic low-level inflammation was sustained into late-life, in turn leading to H2AX degradation and a lack of γH2AX reactivity in these cases. H2AX deficiency has also been linked to chronic neurobehavioral symptoms and an impaired ability to respond to ROS [167], which could further contribute to symptoms and pathology in individuals despite not being reactive for γH2AX. Indeed, gene expression analysis with NanoString of cases without apparent DNA damage as shown by γH2AX revealed up-regulation of pathways involved in cellular senescence [82], supporting the finding that senescent cells are present in mTBI brains even without γH2AX reactivity.

### Nuclear structural changes

Additionally, mTBI brains revealed loss of lamin B1 and H3K27Me3 expression in glial cells consistent with reports on senescence. Lamin B1 normally functions to tether heterochromatin to the inner nuclear membrane, preventing its transcription [15]. However, in senescent cells lamin B1 expression is reduced [40], resulting in rearrangement of heterochromatic regions into senescence-associated heterochromatic foci (SAHF) and large-scale changes in gene expression [138]. In addition to lamin B1, senescent cells show a reduction in H3K27Me3, a tri-methylated histone which plays various physiological roles in development, proliferation, and embryonic stem cell differentiation. Loss of the trimethylation status of H3K27Me3 has been reported to induce senescence through the up-regulation of SASP and p16 pathways, and its loss/decrease is therefore considered a marker of senescent cells [64, 66, 170]. Loss of these nuclear proteins has been linked to neurodegeneration and cognitive decline, the details of which will be discussed below.

### mTBI and impairment of DNA repair pathways

Consistent with studies showing that transcriptional repression of DNA repair genes is a hallmark of senescence [49], we saw a general trend of decreased expression in mTBI brains compared to controls with 36 genes being significantly down-regulated and only 9 genes significantly up-regulated. Knocking down specific DNA repair genes is sufficient to induce premature senescence in vivo [22], indicating a role of inefficient DNA repair in the emergence of cellular senescence and subsequent neurological dysfunction in mTBI. The mechanism by which DNA repair genes are down-regulated in association with cellular senescence, is not entirely clear, but may be due to chronically increased levels of oxidative stress associated with a pro-inflammatory toxic environment [49]. In a study on hyperglycemia, for example, it was found that exposure to high levels of glucose resulted in increased levels of ROS in hepatocytes [122]. Initially, this led to increased expression of DNA repair genes, however long-term exposure eventually led to the reduced expression of DNA repair genes and subsequent accumulation of DNA damage [122]. Decreased levels of DNA repair genes have also been reported in AD brains. Indeed, a reduction in the DNA repair protein breast cancer type 1 susceptibility protein (BRCA1) expression has been reported in the brains of human AD patients as well as an amyloid mouse model of AD, and depletion of BRCA1 is suggested to contribute to the cognitive decline seen in AD [148]. In fact, reduced expression of DNA repair genes due to increased levels of oxidative stress has been implicated with several other human diseases, including diabetes [84], obesity [137], and glaucoma [109]. This study suggests that some individuals may be susceptible to the long-term effects of mTBI due to their inability to effectively repair DNA. On the other hand, some individuals may be more resilient to the long-term effects of mTBI due to efficient DNA repair pathways. Indeed, genetic polymorphisms in genes encoding DNA repair proteins may lead to more or less efficient DNA repair and therefore confer, respectively, a decreased or an increased risk of suffering long-term symptoms and neurodegenerative disease after mTBI. In particular, it is possible that inefficient DNA repair pathways underlie sex differences in the experience of concussion. Following mTBI, women tend to experience more symptoms and take significantly longer to recover compared to men for reasons currently unknown [28, 51, 67, 114]. It is known that estrogen and its downstream metabolites induce DNA damage, in DNA stretches that are specifically repaired by BRCA1 [133]. Furthermore, ovariectomy in female rats has been shown to protect against hippocampal DNA damage and improve memory function [85]. Together this indicates that accumulation of sex hormone-induced DNA damage due to inefficient DNA repair can have lasting effects on the brain. Thus, women face additional sources of DNA damage compared to men, and an inefficient DNA repair response may exacerbate this discrepancy. We therefore postulate that inefficient DNA repair machinery after mTBI, as shown in this study, may underlie the substantial sex differences in the experience of mTBI. Further research regarding the role of specific DNA repair genes in conferring susceptibility or resilience to mTBI, both between individuals and between sexes, will be helpful in clarifying specific polymorphisms in conferring resilience or susceptibility to mTBI.

### The mechanistic action of cellular senescence: shift of glial cellular function

mTBI brains showed substantial morphological changes reflective of cellular senescence. Astrocytic cell bodies in the isocortex of mTBI brains were abnormally swollen and enlarged. Furthermore, sub-pial astrocytes in mTBI brains with DNA damage presented with beading of axonal processes (Figs. 10 and 11a-d). In contrast, brains without a history of trauma and with no DNA damage had normal astrocytic processes (Fig. 11d-e). This is a hallmark of senescence in astrocytes, suggesting that these cells have taken on a secretive function [25]. Cellular senescence is detrimental to overall brain health, through its various effects on different cell types [19]. For instance, when astrocytes become senescent they no longer provide trophic support to the neuron, disturbing normal neuronal function [43]. Astrocytes comprise the majority of all brain cells, and are immediate responders to brain trauma, infections, and neurodegeneration [142]. Indeed, in response to various stressors such as hydrogen peroxide [9] and ionizing radiation [173], astrocytes become senescent. Furthermore, astrocytic senescence has been reported in brains of aged animals [118, 124], indicating that there is a correlation between brain ageing and astrocytic senescence. In the context of traumatic brain injury, post-mortem human brains after blast exposure show astrogliosis in sub-pial regions, specifically surrounding the grey-white matter junctions, in the subpial area and lining of ventricles [139]. The morphology of astrocytes in these cases was similar to the cases illustrated here, in which astrocytic cell bodies are swollen and cellular processes are beaded (see figure 1 in reference [139]). Furthermore, astrogliosis in these individuals was thought to be the basis of post-traumatic stress disorder (PTSD) in soldiers who had suffered blast exposure-related mTBI [139]. Other important brain cells affected by senescence are oligodendrocytes, which may lose their ability to myelinate and therefore disrupt axonal health and signaling capabilities when they become senescent [154]. Microglia also can become senescent, which affects their ability to mediate immune responses in the CNS in response to injury or infection [23]. Indeed, microglia from aged human brains are generally swollen and show fragmentation of processes thought to contribute to neuronal death [38]. Lastly, senescent endothelial cells may disrupt the integrity of the blood-brain barrier [1], an effect which has been associated with age-related cognitive decline [150]. Cellular senescence in glial cells therefore has vast repercussions on the integrity of neuronal function and global brain health, resulting in widespread tissue dysfunction and, inevitably, the emergence of neurological symptoms. The role of senescent cells in cognitive decline and p-tau pathology has been demonstrated in a transgenic mouse model of AD, in which eliminating senescent cells through senolytic intervention resulted in reduced tau phosphorylation, improved cognitive outcomes, and the prevention of the upregulation of senescence genes [14]. In the context of TBI, markers of senescence have been shown to elevate in microglia and astrocytes following a controlled cortical impact protocol [153]. Contrary to studies showing vascular dysfunction in mTBI [2, 157], we did not see evidence of cellular senescence in endothelial cells. Furthermore, we did not specifically evaluate senescent microglia in this cohort. Future studies utilizing double-labeling techniques would help clarify the involvement of endothelial cells and microglia in mTBI-related senescence. Cellular senescence is therefore a powerful mechanism affecting mainly glial cells and capable of inducing overall brain dysfunction, in many instance without visible structural damage on histological examination, and leading to important neurocognitive deficits.

We suggest that senescent glial cells have detrimental effects on the function of neurons, and we have shown some preliminary markers of neuronal dysfunction. It is well known that glial cells are critical support cells for neurons [159], and that their loss can induce neuronal dysfunction [72]. In this study, we have revealed several changes in neurons suggesting changes in genome integrity, nuclear membrane structure, and axonal signalling. Indeed we found that cases with senescence presented with loss of nuclear proteins BRG1 and intranuclear tau. BRG1 is a transcription factor critical for healthy neuronal gene expression and functioning, including neuronal differentiation and the function of synapses [97]. In studies on ALS for example, loss of crucial BRG1 subunits was found to cause dendritic attrition, which was delayed by overexpressing BRG1 [152]. Furthermore, mutations in BRG1 led to reduced dendritic spine density, impaired synapse activity, and neurological deficits in a study on autism spectrum disorder [171]. Together, the literature indicates that loss of BRG1 expression is an important marker of neuronal function, and may have implications for clinical manifestation. In addition to BRG1, we report translocation of neuronal intranuclear tau protein to the cytoplasm in cases with evidence of glial senenescence compared to controls. Tau protein, most well-known as a structural molecule of the cytoskeleton in the axons and for its involvement in AD pathogenesis in its hyperphosphorylated form, has been found to be normally expressed within the nucleus of neurons [12]. Intranuclear tau has been shown to interact with nucleic acid proteins and other nuclear proteins, and various studies ranging from cell culture to human brain have suggested that it is essential for genome integrity [12]. In particular, intranuclear tau is thought to bind to chromatin and stabilize it in response to cellular stressors such heat [149]. A recent study on post-mortem AD brains revealed translocation of intranuclear tau to the soma in diseased brains compared to controls [57], indicating that intranuclear tau may also play a role in its pathogenesis. In addition to changes in genome integrity, we found loss of the nuclear envelop protein emerin, an integral protein of the nuclear membrane which functions to tether chromatin and help stabilize the nuclear component, in cases with glial cell DNA damage. Loss of emerin can lead to disruption of signaling pathways critical for maintaining normal transcription [78] and implies disruption of healthy structural integrity of the nuclear membrane. Lastly, we showed loss of MBP expression in neuronal axons and pallor of the white matter, yet intact neurofilament protein. These results suggest that neuronal axonal structure is maintained, but their myelination may be disrupted. We suggest that individuals with damage in oligodendrocytes may have a decreased ability to myelinate neuronal axons, potentially leading to disruption in neuronal communication. A significant decrease in myelination, even in small regions, may be sufficient to disrupt communications through large networks and therefore possibly lead to neurological dysfunction. Although these results are fairly preliminary, we believe that senescent glial cells may impact neuronal functioning such that their basic properties, namely genome integrity, nuclear membrane structure, and communication between cells and networks, may be disrupted. Further studies using experimental models will be critical in understanding the effects of glial cell senescence on neuronal function, and the present study should be considered an exploration into the neuronal involvement in glial cell senescence.

### Cellular senescence and neurodegenerative disease

Nearly every case presented was symptomatic, but several cases did not show any abnormal neuropathological changes. In contrast, DNA damage and evidence of cellular senescence was widespread, leading us to question whether cellular senescence may be the driver of symptoms in these cases rather than protein aggregation. Indeed, cellular senescence has been suggested as a contributing factor causing neurodegenerative pathology [143], and has been hypothesized to be involved in AD pathogenesis [46]. Recently, the assumption that pathological protein burden reflects clinical presentation in neurodegenerative diseases has been called to question [35]. There is now evidence showing that equivalent loads of AD-consistent pathology in different cases can be associated with various cognitive outcomes [63]. Indeed, individuals with the same load of beta-amyloid and p-tau pathology can present anywhere on a spectrum from cognitively intact to demented [111]. This points to the idea that abnormal protein deposits in the brain may not be the only explanation of symptoms, and that perhaps other unknown molecular mechanisms are driving brain dysfunction, leaving protein deposits as an end-point process. A failure of anti-amyloid therapies, such as AN1792 [45] and solanezumab [33], in producing clinical benefits in AD experimental trials further supports the possibility that proteinopathy burden, in AD in this particular example, may not explain clinical symptoms. Another exemplary phenomenon is age-related tau astrogliopathy (ARTAG), in which p-tau accumulates in astrocytes of various brain regions [79]. ARTAG is often seen in individuals over the age of 60 who, despite accumulating p-tau, do not present with any clinical symptoms [80]. This phenomenon therefore highlights the frequent discordance between pathological load and clinical symptoms. In light of these discrepancies, we therefore suggest that cellular senescence represents a plausible pathophysiological mechanism which can contribute to symptoms and possibly drive neurodegenerative disease in the context of mTBI.

Although this study did not directly assess the role of cellular senescence in the emergence of neuropathology, it is possible that the acquisition of cellular senescence after mTBI renders brains more susceptible to neurodegenerative disease through a mechanism yet to be unraveled. We explored the relationship between γH2AX and proteinopathies and found that in controls with no history of mTBI and no proteinopathy, DNA damage was not present. In contrast, mTBI cases frequently had evidence of DNA damage even in the absence of a proteinopathy. Indeed, cases with no proteinopathy tended to have lower stages of γH2AX (stage 0–2) compared to cases with more severe proteinopathies, which ranged from low to high stages of γH2AX (stages 0–3). Although, as discussed above, low stages of γH2AX confined to the ependymal lining, may have significantly larger effects due to involvement of paracrine signalling in the CSF. When we further stratified our cases by the level of tau burden, we found that mTBI cases with mild and severe tauopathy tended to have higher levels of DNA damage than mTBI cases with no tauopathy, and that controls with no mTBI history and no tauopathy did not have any DNA damage. This relationship was not statistically significant, but it suggests a trend of increased burden of tau with increased DNA damage. The precise relationship between DNA damage-induced cellular senescence, tau burden, and symptoms is not known, but we can speculate several contributing factors to neuropathology in this cohort. One possible driver of tauopathy is the activation of the DNA damage response. In particular, DDR proteins CHEK1 and CHEK2 have been shown to phosphorylate tau protein, and contribute to its accumulation [65]. In this cohort, mTBI brains displayed significant upregulation of both CHEK1 and CHEK2 expression compared to controls. Another important target of CHEK2 phosphorylation is p53 [169], a critical transcriptional regulator of cellular senescence [130]. Upregulation of CHEK1 and CHEK2 may therefore cause both increased p-tau burden and accumulation of senescent cells. Another factor to consider is the degradation of H2AX. Indeed, because some mTBI cases with severe proteinopathy presented with no DNA damage this reinforces the previously discussed notion that perhaps γH2AX is degraded in some individuals with chronic history of head trauma, and that this depletion may contribute to the emergence of neurological symptoms. Lastly, most cases presented with loss of lamin B1, indicating the acquisition of cellular senescence in these cells. Loss of lamin B1 has been associated with cognitive decline and neurodegenerative pathology [20, 56]. Indeed, AD has been explicitly referred to as an acquired neurodegenerative laminopathy [41], and lamin dysfunction has been shown to drive tau-mediated neurodegeneration [42]. In summary, even in cases with no evidence of DNA damage, symptoms and pathology associated with mTBI may emerge due to upregulation of the DDR, the degradation of H2AX, and/or the loss of lamin B1 attributed to senescence. It is quite possible that a combination of these molecular changes contribute to neurodegenerative pathology and symptomology associated with mTBI. The interaction of these multiple factors may even contribute to the heterogeneity in pathological outcomes associated with mTBI. As we have described, the neuropathology of mTBI is quite complex and reflects several different diseases including AD, PD, FTD, ALS, and CTE. Indeed, we propose that inefficient DNA repair and subsequent DNA damage-induced cellular senescence precedes neuropathological changes, and renders mTBI brains susceptible to neurodegenerative pathology.

## Supplementary information

**Additional file 1.** List of 169 genes included in NanoString custom panel.

## Data Availability

The data generated or analysed during this study are primarily included in this published article and any additional information may be available from the corresponding article upon reasonable request.
