# Supplementary material for: DNA repair deficiency and senescence in concussed professional athletes involved in contact sports
Source: Acta Neuropathol Commun. 2019 Nov 14;7:182. doi: 10.1186/s40478-019-0822-3 (PMC6857343; doi:10.1186/s40478-019-0822-3)
Supplement: Supplementary file 1 — Additional file 1. List of 169 genes included in NanoString custom panel. [file 40478_2019_822_MOESM1_ESM.docx]

| Gene Symbol | Gene Name |
| --- | --- |
| APEX 1  APEX 2  ATM  ATR  ATRIP  BARD1  BLM  BMI1  BRCA1  BRCA2  BRIP1  CCL1  CCL11  CCL13  CCL16  CCL2  CCL20  CCL25  CCL26  CCL28  CCL3  CCL4  CCL7  CCL8  CCNH  CDK2  CDK7  CETN2  CHEK1  CHEK2  CITED2  CXCL1  CXCL11  CXCL12  CXCL2  CXCL3  CXCL5  CXCL6  CXCL8  CXCR2  DDB1  DDB2  DMC1  ERCC1  ERCC2  ERCC3  ERCC4  ERCC5  ERCC6  ERCC8  ETS1  EXO1  FANCA  FANCD2  FANCG  FEN1  GADD45A  GADD45B  GADD45G  GFAP  GSK3B  GTF2H1  H2AFX  HMGB1  HUS1  ID1  IFNG  IL10  IL11  IL12A  IL12B  IL2  IL4  IL6  IL7  IL6R  LIG1  LIG3  LIG4  MAG1  MAP3K13  MBD4  MCPH1  MDC1  MDM2  MGMT  MIF  MLH1  MLH3  MMS19  MNAT1  MPG  MSH2  MSH3  MSH4  MSH5  MSH6  MTOR  MUTYH  NBN  NEIL1  NEIL2  NEIL3  NFATC1  NFATC2  NFKB1  NOX4  NRF2  NTH1  OGG1  PARP1  PARP3  PCNA  PMS1  PMS2  PNKP  POLB  POLD1  POLD2  POLD3  POLL  PPP1R15A  PRKDC  RAD1  RAD17  RAD18  RAD21  RAD23A  RAD23B  RAD50  RAD51  RAD51B  RAD51C  RAD51D  RAD52  RAD54L  RAD9A  RELA  RNF168  RNF8  RPA1  RPA2  SERPINB2  SERPINE1  SMUG1  SUMO1  TDG  TERF2  TERT  TNFRSF14  TNFRSF18  TP53  TP53BP1  TP73  TREX1  UNG  XAB2  XPA  XPC  XRCC1  XRCC2  XRCC3  XRCC4  XRCC5  XRCC6 | Apurinic/Apyrimidinic Endodeoxyribonuclease 1  Apurinic/Apyrimidinic Endodeoxyribonuclease 2  Ataxia Telangiectasia Mutated Serine/Threonine Kinase  Ataxia Telangiectasia And Rad3-Related Protein Serine/Threonine Kinase  ATR Interacting Protein  BRCA1 Associated RING Domain 1  Bloom Syndrome RecQ Like Helicase  Polycomb Group RING Finger Protein 4  Breast Cancer Type 1 Susceptibility Protein  Breast Cancer Type 2 Susceptibility Protein  BRCA1 Interacting Protein C-Terminal Helicase 1  Chemokine (C-C Motif) Ligand 1  Chemokine (C-C Motif) Ligand 11  Chemokine (C-C Motif) Ligand 13  Chemokine (C-C Motif) Ligand 16  Chemokine (C-C Motif) Ligand 2  Chemokine (C-C Motif) Ligand 20  Chemokine (C-C Motif) Ligand 25  Chemokine (C-C Motif) Ligand 26  Chemokine (C-C Motif) Ligand 28  Chemokine (C-C Motif) Ligand 3  Chemokine (C-C Motif) Ligand 4  Chemokine (C-C Motif) Ligand 7  Chemokine (C-C Motif) Ligand 8  Cyclin H  Cyclin-Dependent Kinase 2  Cyclin-Dependent Kinase 7  Centrin 2  Checkpoint kinase 1  Checkpoint kinase 2  Carboxy-terminal domain 2  C-X-C Motif Chemokine Ligand 1  C-X-C Motif Chemokine Ligand 11  C-X-C Motif Chemokine Ligand 12  C-X-C Motif Chemokine Ligand 2  C-X-C Motif Chemokine Ligand 3  C-X-C Motif Chemokine Ligand 5  C-X-C Motif Chemokine Ligand 6  C-X-C Motif Chemokine Ligand 8  C-X-C Motif Chemokine Receptor 2  Damage specific DNA binding protein 1  Damage specific DNA binding protein 2  DNA meiotic recombinase 1  Excision Repair Cross-Complementation Group 1  Excision Repair Cross-Complementation Group 2  Excision Repair Cross-Complementation Group 3  Excision Repair Cross-Complementation Group 4  Excision Repair Cross-Complementation Group 5  Excision Repair Cross-Complementation Group 6  Excision Repair Cross-Complementation Group 8  ETS Proto-Oncogene 1 Transcription Factor  Exonuclease 1  Fanconi anemia complementation group A  Fanconi anemia complementation group D2  Fanconi anemia complementation group G  Flap structure-specific endonuclease 1  Growth arrest and DNA damage inducible alpha  Growth arrest and DNA damage inducible beta  Growth arrest and DNA damage inducible gamma  Glial fibrillary acidic protein  Glycogen synthase kinase 3 beta  General transcription factor IIH Subunit I  H2A histone family member X  High mobility group box 1  Checkpoint protein HUS1  Inhibitor of DNA binding 1, HLH protein  Interferon gamma  Interleukin 10  Interleukin 11  Interleukin 12A  Interleukin 12B  Interleukin 2  Interleukin 4  Interleukin 6  Interleukin 7  Interleukin 6 receptor  DNA ligase 1  DNA ligase 3  DNA ligase 4  DNA-3-methyladenine glycosylase  Mitogen-activated protein kinase kinase kinase 13  Methyl-CpG binding domain 4, DNA glycosylase  Microcephalin 1  Mediator of DNA damage checkpoint 1  MDM2 proto-oncogene  O-6-methylguanine-DNA methyltransferase  Macrophage migration inhibitory factor  MutL homolog 1  MutL homolog 3  MMS19 homolog, cytosolic iron-sulfur assembly component  MNAT, CDK activating kinase assembly factor  N-methylpurine DNA glycosylase  MutS homolog 2  MutS homolog 3  MutS homolog 4  MutS homolog 5  MutS homolog 6  Mechanistic target of rapamycin kinase  MutY DNA glycosylase  Nirbin  Nei like DNA glycosylase 1  Nei like DNA glycosylase 2  Nei like DNA glycosylase 3  Nuclear factor of activated T cells 1  Nuclear factor of activated T cells 2  Nuclear factor kappa beta subunit 1  NADPH oxidase 4  Nuclear factor, erythroid 2 like 2  NTH like DNA glycozylase 1  8-oxoguanine DNA glycosylase  Poly(ADP-ribose) polymerase 1  Poly(ADP-ribose) polymerase 3  Proliferating cell nuclear antigen  PMS1 Homolog 1, mismatch repair system component  PMS2 Homolog 2, mismatch repair system component  Polynucleotide kinase 3’-phosphatase  DNA polymerase beta  DNA polymerase delta 1, catalytic subunit  DNA polymerase delta 2, accessory subunit  DNA polymerase delta 3, accessory subunit  DNA polymerase lambda  Protein phosphatase 1 regulatory subunit 15A  Protein kinase, DNA-activated, catalytic subunit  RAD1 checkpoint DNA exonuclease  RAD17 checkpoint clamp loader component  RAD18, E3 ubiquitin protein ligase  RAD 21 cohesin complex component  RAD23 homolog A, nucleotide excision repair protein  RAD23B homolog B, nucleotide excision repair protein  RAD50 double strand break repair protein  RAD51 recombinase  RAD51 paralog B  RAD51 paralog C  RAD51 paralog D  RAD52 homolog, DNA repair protein  RAD54 like  RAD9 checkpoint clamp component A  RELA proto-oncogene, NFKB subunit  Ring finger protein 168  Ring finger protein 8  Replication protein A1  Replication protein A2  Serpin family B member 2  Serpin family E member 1  Single-strand selective monofunctional uracil-dna glycosylase 1  Small ubiquitin-like modifier 1  Thymine DNA glycosylase  Telomeric repeat binding factor 2  Telomerase reverse transcriptase  TNF receptor superfamily member 14  TNF receptor superfamily member 18  Tumor protein 53  Tumor protein binding protein 1  Tumor protein p73  Three prime repair exonuclease 1  Uracil DNA glycosylase  XPA binding protein 2  XPA, DNA damage recognition and repair factor  XPC complex subunit, DNA damage recognition and repair factor  X-Ray repair cross complementing 1  X-Ray repair cross complementing 2  X-Ray repair cross complementing 3  X-Ray repair cross complementing 4  X-Ray repair cross complementing 5  X-Ray repair cross complementing 6 |
